# Mindfulness for the self‐management of negative coping, rumination and fears of compassion in people with cancer: An exploratory study

**DOI:** 10.1002/cnr2.1761

**Published:** 2022-12-27

**Authors:** Sian Williams, Seraphine Clarke, Trudi Edginton

**Affiliations:** ^1^ Department of Psychology City, University of London London UK

**Keywords:** cancer, compassion, coping, depression, mindfulness, ruminating

## Abstract

**Background:**

Cancer and its treatments have the potential to significantly impact mental health, provoking feelings of anxiety, depression, and distress, which can last long after treatment is over. One of the most rapidly emerging influences in the healthcare field is mindfulness‐based interventions (MBIs), which are designed to cultivate present moment awareness, attentional flexibility, compassion and acceptance, to reduce physical and psychological distress. However, there is limited research into the efficacy of MBIs or disease specific MBIs in shifting negative coping, ruminative thinking and fears of compassion as primary outcomes in individuals with cancer.

**Aims:**

This exploratory study was designed to evaluate inter‐ and intra‐individual change in the management of negative coping, rumination and fears of compassion, following a cancer‐specific mindfulness‐based intervention.

**Methods and Results:**

A single group, non‐experimental, repeated measures study of 22 participants across six cancer care centres explored the efficacy of an 8‐week Mindfulness‐Based Cognitive Therapy for Cancer (MBCT‐Ca) course. The Reliable Change Index (RCI) examined reliable clinical improvement, deterioration, or no change in individuals on the Mental Adjustment to Cancer Scale (MACS), the Ruminative Responses Scale (RRS) and the Fears of Compassion Scale (FCS). About 82% of participants (*n* = 18) saw an improvement in at least one measure. A significant decrease in primary outcome scores was observed in negative coping, ruminating and fears of self‐compassion. There were significant correlations between the fear of self‐compassion and depressive ruminating, fear of accepting compassion from others and showing it to others pre and post intervention.

**Conclusion:**

Our findings indicate that the MBCT‐Ca programme may significantly reduce negative coping, ruminating and fears of self‐compassion improving psychological health and wellbeing in cancer survivors.

## 1 INTRODUCTION

Cancer and its treatments do not just impact physical health and everyday functioning, they have the potential to significantly affect mental health and psychological wellbeing too, provoking feelings of anxiety, depression, and distress which can last long after treatment is over.^1^, ^2^ Individuals can experience the fear of disease recurrence more than 5 years post‐treatment^3^ and subsequent unresolved mental health issues in cancer survivors are known to lead to more frequent doctor and hospital visits, with associated increases in social and health care costs.^4^

Evidence‐based group mindfulness‐based interventions (MBIs) have emerged as a viable and cost‐effective option for a range of clinical conditions including cancer, chronic pain, depression, stress and anxiety, but mindfulness can often be misunderstood, difficult to define and hard to measure systematically due to its experiential nature and its many adaptations and approaches.^5^, ^6^ There is sufficient consensus, however, to suggest that the mechanisms of mindfulness encourage the individual to shift their perspective on thoughts, emotions, and sensations so that rather than ruminating over them, they are held in a non‐judgemental, moment‐to‐moment place, encouraging awareness, equanimity and openness through intention, attention, and attitude.^7^, ^8^

The most widely researched MBI is Mindfulness‐Based Stress Reduction (MBSR)^9^, ^10^ a structured 8‐week group programme of mindfulness, gentle yoga, and body scans with additional home practice. Mindfulness‐Based Cognitive Therapy (MBCT) is an adaptation of MBSR and combines the MBSR curriculum with cognitive behavioural therapy techniques.^11^ Multiple research studies have confirmed MBCT efficacy in managing the risk of relapse in depression compared to treatment as usual.^12^, ^13^, ^14^ and it is recommended by the UK's National Institute for Health and Care Excellence (NICE) as a priority treatment for depression.^15^, ^16^

Traditionally, the mechanisms of change associated with MBIs have focused on equanimity, cognitive defusion, attentional flexibility and acceptance, that decouple the emotional salience and negative thinking seen in individuals suffering from depression,^17^ which feels particularly relevant to those living with cancer.^18^ Cancer survivors often experience emotion regulation difficulties, and the presence of low mood and ruminative thinking, together with lower levels of self‐compassion and motivation, can impact on physical, emotional, and cognitive function.^19^ Systematic reviews, meta‐analyses, and individual studies into MBSR and/or MBCT for cancer survivors reveal beneficial effects in reducing depression, anxiety and stress, leading to an improvement in quality of life.^20^, ^21^ Research into the efficacy of MBSR and breast cancer suggests participants reported an increased ability to cope, manage and find meaning^22^ and a reduction in fear of recurrence.^23^ In addition, a systematic review and meta‐analysis of MBSR studies in a broader cancer population reported evidence for reliable improvements in reducing individuals' psychological distress.^24^

The robust and consistent effects of MBCT and MBSR have been established for individuals with cancer however, there is less research into programmes specifically designed for individuals with the disease, such as Mindfulness‐Based Cancer Recovery (MBCR)^25^ and MBCT for Cancer (MBCT‐Ca).^26^ One study suggests MBCR is superior to supportive‐expressive group therapy in reducing mood disturbance and stress symptoms^27^ and a study of MBCT‐Ca suggests improvements in depression and quality of life.^28^ However, to the authors' knowledge, there is currently no published study into the impact on ruminating and fears of compassion, after a cancer‐specific mindfulness‐based intervention.

Rumination is defined as attending to intrusive negative thoughts repeatedly, and fits broadly into three categories; brooding rumination, thought to be excessive and non‐productive; depressive rumination, characterised as a focus on one's feelings of sadness; and reflective rumination, defined as being more purposeful and problem‐solving.^29^ The idea of self‐compassion as a buffer against adversity is relatively new in psychopathology, but research in those with breast cancer suggests that it activates the resting parasympathetic nervous system and suppresses the threat system, to lower rumination and anxiety.^30^ Interestingly, the differentiate with this study proposed that mindfulness was instrumental as a mechanism of change for the augmented self‐compassion and lowered rumination. However, one study suggests low self‐worth, negative emotions, suppression of painful thoughts and an anxious or avoidant attachment style can make engaging with mindfulness and developing self‐compassion difficult for individuals with cancer and it recommended further research into interventions which focus on self‐kindness to improve psychological outcomes for these patients.^31^

Despite the range of physical and psychological benefits of mindfulness‐based interventions, there can be limitations to mindfulness research in those with cancer. A systematic review of MBIs and RCTs in cancer cohorts suggested most interventions were variable and poorly defined, that the protocol often changed during treatment and that information was gathered sometime after the MBI had been delivered^32^ In addition, the possibility of a high risk of performance and detection bias has also been reported in some patient cohorts.^33^ A review of 124 RCTs in MBIs in healthcare, concluded that almost 90% reported positive results and the authors suggest this may be due to effect sizes being over‐stated, selective outcome reporting, ‘data dredging’ and overall reporting bias.^34^

Any intervention that has the potential for relieving psychological distress, also risks adverse effects^35^ and there are a small number of studies reporting negative outcomes in mindfulness.^36^ One study with chemotherapy patients suggested an increase in symptom distress and reduced quality of life, compared to those following a relaxation programme.^37^ However, this was based on three 90‐min sessions of MBCT‐Ca and did not follow its established protocol. A recent review of the potential for harm in MBIs suggests this and other studies should be seen in the context of meta‐analyses suggesting positive benefits for patients, including those with cancer.^38^ This is not to say adverse events and negative experiences do not happen, but as most research examines group averages, meaningful deterioration in participants may be masked. The authors of the review call for researchers to examine individual‐level data and suggest using the Reliable Change Index (RCI), which measures clinically significant improvements, deterioration, or no change in each participant.^39^

Mindfulness should not be seen as a cheap option, it is not risk‐free, nor appropriate for all, however as a targeted intervention for those struggling with the emotional effects of cancer, it can help develop psychological flexibility, wellbeing, and self‐compassion. This study has been designed to assess the inter‐ and intra‐individual impact of an 8‐week MBCT‐Ca programme on those who were either receiving active treatment, recovering from treatment, or in remission from cancer, with a focus on reliable change indices, on an individual level. The primary aim of the study was to examine any shift in mental adjustment to cancer, ruminating and fears of compassion after an intervention tailored to those with the disease. A secondary aim was to explore the potential relationship between these concepts.

## 2 METHODS

This was a single group, non‐experimental, repeated measures exploratory study of participants who had enrolled on an MBCT‐Ca intervention at a UK charity offering free cancer support.

### 2.1 Participants

There were 20 women and two men. All participants were over 45 and 64% (*n* = 14) had University or post‐graduate qualifications. Half worked full‐time, part‐time or were self‐employed (*n* = 11), 36% were retired (*n* = 8) and 9% (*n* = 2) were unemployed or too ill to work. Most identified as white British, 68% (*n* = 15), with 86% (*n* = 19) specifying English as their main language. The majority were living with or beyond breast cancer, with other cancers including uterine/endometrial, ovarian, prostate, bowel, and kidney cancer. The stages ranged from stage 0 where the cancer is small and contained to stage 4, where it has spread from its origin to another organ. Some participants were in active treatment, others were not (Table 1).

**TABLE 1:** Participants' cancer, stage and treatment

Characteristic	Number (*n*)	Percentage (%)
Cancer diagnosis		
Less than 12 months	8	36
13–24 months	7	32
2–5 years	2	9
More than 5 years	5	23
Stage of cancer		
Stage 0	1	4.5
Stage 1	6	27
Stage 2	4	18
Stage 3	6	27
Stage 4	3	13
Missing	2	9
Type of cancer		
Breast	14	64
Blood	2	9
Uterine/endometrial	2	9
Bowel	1	4.5
Kidney	1	4.5
Ovarian	1	4.5
Prostate	1	4.5
Treatment		
Surgery	18	82
Radiotherapy	13	59
Chemotherapy	14	64
Hormonal	17	77
Biological	8	36

Participants were assessed for mental adjustment to cancer, rumination, and fears of compassion pre and post the MBI.

### 2.2 Sample

Between October and December 2019, six centres running MBCT‐Ca interventions consented to participate in the study. The inclusion criteria, developed in accordance with the MBCT Implementation Resources for recruitment,^40^ included those living with or beyond cancer who were between 25 and 85 years old and could speak and read English. The exclusion criteria included those experiencing an acute episode of depression or anxiety, and/or who had a mental health diagnosis, were addicted to alcohol or drugs and/or had an additional acute life crisis such as a recent bereavement.

Centre heads and mindfulness teachers were not expected to recruit participants, however, interested individuals were directed to the participant information guidance and questionnaire. While supportive group therapy was considered as an alternative control for comparison, the centres were not running groups and resources were not available to create them A wait‐list control would have been difficult to establish, as there was no guarantee of a later intervention and a passive, or no‐control control group might be considered unethical in clinical populations.^41^

### 2.3 Procedure

Ethical approval was gained from a UK‐based University. Courses began in January 2020 and all participants provided consent to participate prior to the intervention starting. Those providing an email were sent a consent form, participant information letter and a link to the baseline questionnaire, which was developed on Qualtrics, following a pilot study to assess its suitability for individuals already undergoing a clinically difficult experience.^42^ A reminder email was sent towards the end of the course. If the follow‐up questionnaire had not been returned a week after the intervention finished, a further reminder was sent.

All MBCT‐Ca interventions were led by trained mindfulness teachers who followed an established protocol of eight weekly group sessions of around 2.5 hours including meditations, body scans, ‘pause’ exercises and guidance in noticing reactions to difficult or unpleasant experiences (see Appendix for example of session 1 protocol). The course embodies the approaches of MBCT for depression^43^ and MBSR^9^ with a focus on suffering, practice, and presence. There is no yoga element and the cognitive model, explored in week four, investigates responding to distress in the cancer experience. The course includes an all‐day practice in week six and participants are encouraged to practise mindful exercises at home.

### 2.4 Measures

#### 2.4.1 Mental adjustment to cancer, including negative and positive reactions

The Mental Adjustment to Cancer Scale (MACS)^44^ examines reactions and coping strategies. It includes 40 statements on a 4‐point scale from 0 (‘definitely does not apply to me’) to 4 (‘applies to me’). The original MACS has five subscales, measuring fighting spirit, anxious preoccupation, avoidance, helpless‐hopelessness, and fatalism. Watson and Homewood's revised 33‐item, two‐factor structure^45^ was used in this study, with the summary ‘Positive Adjustment’ Scale representing attitudes and actions, such as a desire to carry on, determination and a positive approach (previously fighting spirit) and the summary ‘Negative Adjustment’ Scale representing feelings such as anxiety, anger and hopelessness (previously helplessness/hopelessness, anxious preoccupation, avoidance, and fatalism). Watson and Homewood suggest this analysis of coping responses into two dimensions is pragmatic, and it has acceptable reliability and internal consistency, with Cronbach alpha scores for both, at .84.

#### 2.4.2 Depressive, brooding, and reflective rumination

The Ruminative Responses Scale (RRS)^46^ examines the presence of repetitive depressing, brooding, or reflective thoughts. It is a series of 22 statements on a 4‐point scale, from 1 (‘almost never’) to 4 (‘almost always’) and assesses an individual's cognitive coping style such as entangling with feelings, repeatedly paying attention to symptoms, and/or trying to work out causes or consequences of emotion. It has strong psychometric properties and reliability, with Cronbach alpha scores at or around .90.^47^

#### 2.4.3 Fears of compassion from others, to others and towards the self

The Fears of Compassion scale (FCS)^48^ assesses fears of acknowledging kindness from others, expressing it to them and showing it towards the self. It is a 20‐item scale, and the original study with students and therapists showed acceptable reliability; with .87 for fears of compassion from others, .78 to others and .85 towards the self. The FCS was originally a 5‐point scale with 0 (‘don't agree at all’) to 4 (‘completely agree’). This was the final measure in the questionnaire and a sliding scale was used, as research suggests it can maximise engagement and prevent drop‐off.^49^ As studies suggest participants using sliding scales often choose the default, mid‐point position,^50^ a 6‐point scale was selected, thus increasing reliability, validity and discriminating power.^51^

### 2.5 Statistical analysis

Significance values were set at *p* < .05 and confidence intervals at 95%. Baseline characteristics of the sample were measured using descriptive statistics. Parametric assumptions tested for normality of distribution and multicollinearity using SPSS statistical software. The assumption of normality was satisfied for all subscales of the MACS and RRS in both pre‐ (T1) and post‐ (T2) questionnaires. The changes in scores were analysed using a two‐tailed, paired samples *t*‐test. The non‐parametric Wilcoxon Signed Ranks Test was used to assess changes in the FCS where normality was violated. It is a sensitive, flexible statistical test for non‐normal data distribution and is used widely in healthcare research.^52^

A Pearson correlation analysis evaluated the association between mental adjustment to cancer, ruminative thinking, and fears of compassion both before and after the course. All scales and subscales appeared to have good internal consistency in both pre‐ and post‐questionnaires, with a range of between *a* = .75 and .95.

## 3 RESULTS

Comparisons of available demographic data of those with cancer in a study evaluating the effectiveness of mindfulness on well‐being in a similar setting, suggest the sample was comparable.^53^ Preliminary analysis of the scored data was conducted before the full analysis, to investigate the assumptions of parametric tests, such as the assumptions of linearity, and parametric data were not violated.

### 3.1 Primary outcomes

#### 3.1.1 Mental adjustment to cancer and rumination

**FIGURE 1:**
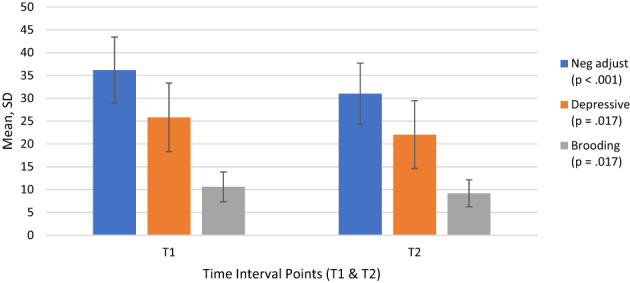
Change in negative adjustment to cancer, depressive and brooding rumination (T1 and T2)

A paired samples *t*‐test compared participants' adjustment to cancer and rumination scores before and after the mindfulness‐based intervention. There were statistically significant differences in the pre‐ (T1) and post (T2) scores for negative adjustment to cancer, and depressive and brooding rumination, although not reflective rumination. Negative adjustment factors, including feelings of helpless/hopelessness, fatalism, anger, denial and anxious preoccupation, were significantly lower after the mindfulness course (*M* = 31.00, SD = 6.71) than before it (*M* = 36.18, SD = 7.24); *t* (21) = 4.68, *p* < .001 and the effect size was large (*d* = 0.7). Depressive rumination scores were significantly lower after the mindfulness intervention (*M* = 22.00, SD = 7.43) than before it (*M* = 25.82, SD = 7.50); *t* (21) = 2.59, *p* = .017 with a medium effect size (*d* = 0.5). Participants also showed lower levels of brooding rumination after the course (*M* = 9.18, SD = 2.95) than before it (*M* = 10.59, SD = 3.27); *t* (21) = 2.58, *p* = .017 with a medium effect size (*d* = 0.5) (Figure 1 and Table 2).

**TABLE 2:** Paired sample *t*‐tests in participants (*N* = 22) adjustment to cancer and rumination (T1 and T2)

	*M* (SD) T1	*M* (SD) T2	MD	*t*	*p*
MACS					
Negative adjustment to cancer	36.18 (7.24)	31.00 (6.71)	5.18	4.68	<.001**
Positive adjustment to cancer	48.36 (5.59)	50.18 (6.14)	2.95	−1.75	.094
RRS					
Depressive rumination	25.82 (7.50)	22.05 (7.43)	3.77	2.59	.017*
Brooding rumination	10.59 (3.27)	9.18 (2.95)	1.40	2.58	.017*
Reflective rumination	10.05 (3.13)	9.55 (3.23)	.50	‐.81	.426

Abbreviations: *M*, mean; MACS, Mental Adjustment to Cancer Scale; MD, mean difference; *p*, significance; RRS, Ruminative Responses Scale; SD, standard deviation; *t*, test score; T1, pre‐outcome (week 0); T2, post‐outcome (week 8).

* *p* = <.05;

** *p* = <.01.

#### 3.1.2 Fears of compassion

Results of the Wilcoxon signed rank test on the Fears of Compassion Scale (FCS) and subscales suggest a statistically significant difference in fears of expressing self‐compassion, which decreased between T1 (*M* = 16.50, SD = 15.13) and T2 (*M* = 13.18, SD = 16.75), with a small effect size (*Z* = −2.019, *p* = .043, *d* = 0.2). This was not the case with fears of responding to the expression of compassion from others (*Z* = −1.31, *p* > .05) or showing it to others (*Z* = −1.04, *p* > .05) (Table 3).

**TABLE 3:** Wilcoxon signed‐rank tests in participants (*N* = 22) regarding fears of expressing compassion for others, from others and for self in T1 and T2

	*M* (SD) T1	*M* (SD) T2	MD	*Z*	*p*
FOCS					
Fear of compassion for others	15.32 (12.53)	14.18 (13.15)	1.14	−1.04	.29
Fear of compassion from others	15.05 (15.23)	12.64 (13.73)	0.5	−1.31	.14
Fear of compassion for self	16.50 (15.13)	13.18 (16.75)	3.21	−2.02	.043*

Abbreviations: FOCS, Fears of Compassion Scale; *M*, mean; *MD*, mean difference; *p*, significance; SD, standard deviation; T1, pre‐outcome (week 0); T2, post‐outcome (week 8); *Z*, test score.

* *p* = <.05; **p = <.01.

#### 3.1.3 Intra‐individual change

The Reliable Change Index (RCI) computes the standard error of measurement and the standard error of difference scores to calculate reliable change in each participant that is not likely due to an error of measurement alone (*p* = <.05). Jacobson and Truax^39^ suggest using the following calculation:

$$\mathrm{RCI}=\frac{\chi^{2}-\chi^{1}}{S_{\text{diff}}},\;\text{Where}\;S_{\text{diff}}=\sqrt{2}{\left(\mathrm{SE}\right)}^{2}\;\text{and}\;\mathrm{SE}=\mathrm{SD}\sqrt{1-\alpha }$$

Formula key: *x* ^1^ = mean score T1. *x* ^2^ = mean score T2. *S*_*diff*_ = standard error of difference. SE = standard error of measurement. SD = standard deviation at T1 (baseline). RCI = Reliable Change Index.

The RCI uses the test–retest reliability of each scale and the standard deviation of the reference, or baseline (T1) measurement, to compute an individual's standardised score and determine whether the difference in scores is in =/−5% area of error distribution. Based on this calculation, 82% of participants (*n* = 18) reported clinically reliable change (RC) in at least one measure between T1 and T2, such as significant decreases in negative adjustment to cancer (27%), depressive (32%) and brooding (23%) rumination and fears of compassion for others (9%), from others (14%) and towards the self (23%). Nearly 23% (*n* = 5) also reported clinically reliable increases in scores, including positive adjustment to cancer (9%), depressive (5%) and reflective (5%) rumination and fears of compassion towards the self (5%) (See Supplementary Information).

### 3.2 Secondary outcomes

Results of the Pearson correlations indicated that there was a significant positive association between negative adjustment to cancer and depressive rumination before the mindfulness‐based intervention, *r* (20) = .64, *p* = .001 and after it, *r* (20) = .53, *p* = .010. There was also a moderate positive correlation with brooding rumination before, *r* (20) = .45, *p* = .035 but not after the course, and negative adjustment was negatively associated with a positive adjustment to cancer in both T1, *r* (20) = −.45, *p* = .036 and T2, *r* (20) = −.44, *p* = .037.

Depressive rumination was strongly positively correlated with brooding rumination both before the intervention, *r* (20) = .76, *p* < .001 and after it, *r* (20) = .80, *p* < .001. Brooding rumination was also moderately positively correlated with reflection in T1, *r* (20) = .43, *p* = .044 and T2, *r* (20) = .51, *p* = .14. We also found a highly significant, positive association between reflection and depressive rumination, *r* (20) = .75, *p* < .001, fears of compassion for others, *r* (20) = .61, *p* = .002, from others, *r* (20) = .62, *p* < .001 and towards the self, *r* (20) = .69, *p* < .001 after the course, but not before it. Both before and after the intervention, the fear of expressing self‐compassion was strongly positively correlated with fear of responding to the expression of compassion from others, *r* (20) = .81, *p* < .001 and showing it to them, *r* (20) = .82, *p* < .001 (Tables 4 and 5).

**TABLE 4:** Pearson's correlations in participants (*N* = 22) in T1 (week 0, pre‐intervention)

	Negative	Positive	Depression	Brooding	Reflection	FoC for	FoC from	FoC self
Negative								
Positive	−.45*							
Depression	.64**	−.17						
Brooding	.45*	−.12	.76**					
Reflection	.15	.31	.16	.43*				
FoC for	.28	.06	.53*	.59*	.39			
FoC from	.55*	−.21	.59**	.51*	.28	.83**		
FoC self	.50*	−.19	.60**	.49*	.30	.81**	.83**	

Abbreviations: Brooding, brooding rumination; Depression, depressive rumination; FoC for, fear of compassion for others; FoC from, fear of compassion from others; FoC self, fear of compassion for self; Negative, negative adjustment to cancer; Positive, positive adjustment to cancer.

* *p* = <.05 (two‐tailed);

** *p* = <.01 (two‐tailed).

**TABLE 5:** Pearson's correlations in participants (*N* = 22) in T2 (week 8, post‐intervention)

	Negative	Positive	Depression	Brooding	Reflection	FoC for	FoC from	FoC self
Negative								
Positive	−.44*							
Depression	.53*	−.20						
Brooding	.35	−.21	.80**					
Reflection	.39	.14	.75**	.51*				
FoC for	.23	.13	.76**	.71**	.61**			
FoC from	.39	−.11	.83**	.71**	.62**	.90**		
FoC self	.16	.18	.70**	.65**	.69**	.81**	.82**	

Abbreviations: Brooding, brooding rumination; Depression, depressive rumination; FoC for, fear of compassion for others; FoC from, fear of compassion from others; FoC self, fear of compassion for self; Negative, negative adjustment to cancer; Positive, positive adjustment to cancer.

* *p* = <.05 (two‐tailed);

** *p* = <.01 (two‐tailed).

## 4 DISCUSSION

This exploratory study adds to established research findings in MBSR/MBCT interventions with cancer patients that report improvements in rumination,^54^ self‐compassion^17^ or both.^55^ Significant improvements following the MBCT‐Ca intervention were observed across the primary outcome measures in levels of negative adjustment to cancer, depressive and brooding rumination, and fears of self‐compassion. Unlike many other studies, this research also interrogated improvements on an individual level, with 82% of participants reporting clinically significant change on at least one measure. However, this did not mean negative thoughts or emotions were not present after the mindfulness intervention. Indeed, some individuals saw a rise in depressive and/or brooding rumination and negative responses to cancer, and although only one of these is considered a statistically significant change when using the RCI, the implications for practitioners might be that in assessing for suitability, participants should be asked about their expectations of mindfulness, be fully briefed about the protocol of the course and what to expect, and to be supported by qualified and experienced mindfulness teachers throughout, if adverse effects are experienced.

Existing research suggests some individuals with cancer experience difficulties receiving affiliative emotions such as compassion^48^ and a better understanding of this was suggested by the secondary aim of this study, which examined the relationships between the concepts of coping, rumination, and fears of compassion in cancer after the MBCT‐Ca. There was a strong relationship between fears of self‐compassion and showing compassion to others or accepting it from others pre‐ and post‐intervention, and it was also highly correlated with brooding and depressive ruminating, adding to studies suggesting rumination can contribute to an active resistance to kindness.^56^

There was no statistically significant change for reflective rumination, which is traditionally considered to be more adaptive, a finding that is also consistent with existing studies.^30^ This may be because mindfulness encourages a degree of reflective introspection by turning towards painful thoughts and feelings which some individuals with cancer may find challenging.^57^ The significant positive associations between reflective ruminating with both depressive rumination and fears of self‐compassion post‐intervention suggests that reflection may have contributed to some negative affect, at least in the short‐term, which adds to existing research.^29^ While reflection can lead to productive insights into adversity, it can also be associated with depressive thinking as it can draw individuals into negative ways of thinking and adversely affect mood^58^ and future MBI research in those with cancer may benefit from a further exploration of this.

There are limitations to this study, including the lack of control group and the small sample size (*N* = 22). The numbers of those eligible to participate was potentially limited by the inclusion and exclusion criteria, changes in disease trajectory affecting commitment^59^ and self‐selection, suggesting a potential for low statistical power and a small effect size.^42^ Participants were directed towards information about the study by centre heads and mindfulness teachers, however the coronavirus pandemic impacted the numbers responding. Of the initial 31 participants who successfully filled out the pre‐course questionnaire, six failed to complete the post‐course questionnaire, either because their course was cancelled due to coronavirus or they were ill, and three did not give a reason. This dropout rate is still lower than many studies into mindfulness in those with cancer which suggest attrition rates of around a third or more.^54^, ^60^, ^61^ Despite the authors' attempts to recruit a diverse sample, another limitation of our study was the fact that our participants were predominantly female, middle‐aged, white, with high levels of educational status, which reduces the generalizability of the study. Although this seems typical of many MBIs,^62^, ^63^ future research could explore potential barriers and whether engagement might be increased using other means of delivery, such as web‐based designs. Further studies into the effects of cancer‐specific, mindfulness‐based interventions in a community setting could strengthen these results by repeating it post‐pandemic, with greater resource and with a control group offering an alternative means of psychological support such as counselling or supportive expressive group therapy. Researchers could also visit centres to support the centre leads and engage the communities directly.

These limitations notwithstanding, this study enhances existing research and provides new insights into how MBIs can help cancer patients deal with negative adjustment to the disease, rumination, and fears of self‐compassion. It adds to previous research by being one of the few studies to examine the efficacy of an MBI specifically targeted towards those with the disease (MBCT‐Ca). This study provides additional rigour, by examining reliable improvement and/or deterioration for each individual using RCI that reflects meaningful clinical changes. A review into MBIs suggests they offer promise across the cancer population and the authors urge further qualitative research into different styles and delivery of mindfulness.^64^ Such qualitative studies could bolster these findings by exploring individuals' experience and understanding of complex concepts such as compassion and rumination and examining participants' meaning of the process and mechanisms of change.

## AUTHOR CONTRIBUTIONS

**Seraphine Clarke:** Methodology (supporting); supervision (supporting); writing – review and editing (supporting). **Trudi Edginton:** Conceptualization (supporting); supervision (lead); writing – review and editing (supporting). **Sian Williams:** Conceptualization (lead); data curation (lead); formal analysis (lead); investigation (lead); methodology (lead); project administration (equal); resources (lead); software (lead); validation (lead); visualization (lead); writing – original draft (lead); writing – review and editing (lead).

## ACKNOWLEDGMENTS

The authors would like to thank the Consultant Clinical Lead Psychologist, Lesley Howells at Maggie's Cancer Centre, the centre heads and the mindfulness teachers, who supported this research. Our thanks too, to the participants who engaged with the study, while living with both the uncertainty of cancer and the Covid‐19 pandemic. We would also like to thank friends and colleagues from City, University of London who provided both encouragement and inspiration. Additionally, the lead author, Dr Sian Williams, would like to thank her family for their patience, wisdom and compassion during difficult times. They are true guides and teachers.

## CONFLICT OF INTEREST

The authors have stated explicitly that there are no conflicts of interest in connection with this article.

## Supporting information


**Appendix S1.** Supporting Information

## Data Availability

The data that support the findings of this study are available on request from the corresponding author. The data are not publicly available due to privacy or ethical restrictions.
