# Supplementary material for: Mindfulness for the self‐management of negative coping, rumination and fears of compassion in people with cancer: An exploratory study
Source: Cancer Rep (Hoboken). 2022 Dec 27;6(3):e1761. doi: 10.1002/cnr2.1761 (PMC10026318; doi:10.1002/cnr2.1761)
Supplement: Supplementary file 1 — Appendix S1. Supporting Information [file CNR2-6-e1761-s001.zip › Cancer Reports - Supplementary Information.docx]

**Supplementary information. Reliable Change table showing significant increase/decrease in individual scores**

| Participant | Negative  Adjustment | Positive  Adjustment | Brooding  Rumination | Depressive  Rumination | Reflective  rumination | FoC for others | FoC from others | FoC for self |
| --- | --- | --- | --- | --- | --- | --- | --- | --- |
| 1 | -3.10 |  |  |  |  |  |  |  |
| 2 |  |  |  |  |  |  |  | -2.29 |
| 3 |  |  |  |  |  |  |  |  |
| 4 |  |  |  |  |  | -2.06 |  |  |
| 5 |  |  |  |  |  |  |  | -2.65 |
| 6 | -3.38 |  | -2.48 |  |  | -3.01 | -4.5 | -2.12 |
| 7 |  |  |  |  | -3.16 |  |  |  |
| 8 | -2.81 |  |  |  | 2.25 |  |  |  |
| 9 | -2.81 |  |  | -2.00 |  |  |  | -2.29 |
| 10 |  | 3.31 |  |  |  |  | -2.0 | 4.7 |
| 11 |  |  |  | -5.66 | -2.25 |  |  |  |
| 12 | -3.10 |  |  | -6.00 | -2.25 |  |  |  |
| 13 |  |  |  |  |  |  |  |  |
| 14 |  |  |  |  |  |  |  |  |
| 15 |  |  | -5.45 | -4.33 |  |  |  |  |
| 16 |  |  | -1.98 |  |  |  |  |  |
| 17 |  |  |  | -4.00 |  |  |  |  |
| 18 | -3.66 |  |  | -2.00 |  |  |  |  |
| 19 |  |  |  | 3.66 |  |  |  |  |
| 20 |  |  |  |  |  |  |  |  |
| 21 |  |  | -1.98 | -2.00 |  | -3.1 |  |  |
| 22 |  | 3.86 |  |  |  |  |  |  |

A Reliable Change Index (RCI) is computed by dividing the difference between the pre-treatment and post-treatment scores by the standard error of the difference between the two scores.

If the RCI is **greater than 1.96**, then the difference is reliable, a change of that magnitude would not be expected due to the unreliability of the measure. If the RCI score is 1.96 or less then the change is not considered to be reliable, it could have occurred just due to the unreliability of the measure. RCI = (posttest - pretest) / SE_meas_

**Supplementary information: Participants’ Sociodemographic Characteristics**

| Characteristic | Number *(n)* | Percentage *(%)* |
| --- | --- | --- |
| ***Gender*** |  |  |
| Female | 22 | 91 |
| Male | 2 | 9 |
| ***Age*** |  |  |
| 45-55 | 11 | 50 |
| Over 55 | 11 | 50 |
| ***Ethnicity*** |  |  |
| White British | 15 | 68 |
| Any other white | 5 | 23 |
| Chinese | 2 | 9 |
| ***Main language at home*** |  |  |
| English | 19 | 86 |
| Missing | 3 | 14 |
| ***Employment*** |  |  |
| Full time | 4 | 18 |
| Part time | 5 | 23 |
| Self employed | 2 | 9 |
| Unemployed | 1 | 4.5 |
| Homemaker | 1 | 4.5 |
| Retired | 8 | 36.5 |
| Too ill to work | 1 | 4.5 |
| ***Education*** |  |  |
| Secondary school | 2 | 9 |
| College | 5 | 23 |
| University | 6 | 27 |
| Post-graduate | 8 | 37 |
| Prefer not to say | 1 | 4 |
