# Supplementary material for: Mindfulness for the self‐management of negative coping, rumination and fears of compassion in people with cancer: An exploratory study
Source: Cancer Rep (Hoboken). 2022 Dec 27;6(3):e1761. doi: 10.1002/cnr2.1761 (PMC10026318; doi:10.1002/cnr2.1761)
Supplement: Supplementary file 1 — Appendix S1. Supporting Information [file CNR2-6-e1761-s001.zip › Cancer Reports Appendix.docx]

Appendix: Session One MBCT-Ca Protocol

Key Tasks for Teachers – Week One

*Teacher preparation – a practice of settling and intention before the session starts.*

Beginning the course:

• Ground rules
• Brief grounding exercise

Building the community:

• Introductions and action methods exercise

Personal intentions:

• A guided practice • Intentions letter

The Raisin exercise:

• and dialogue

Week 1 teaching:

- Defining mindfulness
- Exploring the cancer journey and what mindfulness might offer – through the metaphor of Rilke’s *River* Week 1 practice:
- Body Scan and inquiry

Week 1 short practice:

• The Pause

Concluding:

• Week one home practice
• Distributing course materials – workbook and CD1 • Ending – the bells and closing intention

In: Bartley T Mindfulness-based cognitive therapy for cancer: gently turning towards.: 1. ed. Oxford: Wiley-Blackwell.; 2012
